# A Professional and Technical Puzzle

**Published:** 1851-10

**Authors:** 


### ARTICLE XXII.

### *A Professional and Technical Puzzle.*

THE following extract appeared in one of the Cornish newspapers. In a will cause, tried at the late assizes, a surgeon gave the following understandable evidence for the benefit of twelve Cornish jurymen, in relation to the testator's capability of executing a will: "I found him in erysipelatous inflammation, face and scalp of a dusky brown, covered with furfuraceous scales, the result of the peeling of the cuticle; tongue dark, brown, and dry; pulse 120, and thready; slight subsultus jactitation; low muttering delirium; answered when roused, sometimes coherently, sometimes incoherently; he was in a sleepy comatose state, and clearly moribund."

